# Perceptions and acceptability of drone-based larviciding using *Bacillus thuringiensis israelensis* in rice fields in Madagascar, 2022: a qualitative study

**DOI:** 10.1186/s12936-026-05978-x

**Published:** 2026-07-07

**Authors:** Anna Bowen, Haritiana Mendrika Ranaivoharmimina, Hasina Harinjaka Ramiandrisoa, Sarah Zohdy

**Affiliations:** 1 Malaria Branch, Division of Parasitic Diseases and Malaria,, US Centers for Disease Control and Prevention, Atlanta, GA USA; 2 Abt Global., Antananarivo, Madagascar; 3 Ministry of Public Health, National Malaria Program, Antananarivo, Madagascar; 4 Entomology Branch, Division of Parasitic Diseases and Malaria, US Centers for Disease Control and Prevention, Atlanta, GA USA

**Keywords:** *Bacillus thuringiensis israelensis*, Unmanned aerial devices, Larviciding, Malaria

## Abstract

**Background:**

Madagascar’s malaria burden has been increasing for several years despite continued investments in vector control. Supplemental measures to reduce vector populations and outdoor biting, such as larval source management (LSM), may be required in some settings. Madagascar piloted drone-delivered microbial larviciding using *Bacillus thuringiensis israelensis (Bti)* in rice fields in two districts with populations unfamiliar with drones during Feb–Jul 2022.

**Methods:**

We assessed knowledge and perceptions among purposefully selected rice farmers, rice field workers, and community members during focus group discussions (FGDs) in the two districts three to four months after the intervention. In total, 293 people participated in 39 FGDs, which were analyzed using a combined deductive-inductive approach.

**Results:**

Despite initial concerns, both *Bti*-based larviciding and using drones to apply larvicides were ultimately well accepted by rice field owners and workers and community members in rice-growing areas of Madagascar. Key concerns spanned safety of *Bti* for people, livestock, and the environment, and harms related to drones crashing or being used for nefarious purposes. Participants perceived decreases in mosquitoes and malaria and increased rice yield related to the intervention. Receipt of project messaging appeared to differ by demographic group, with rice field workers frequently reporting having missed community meetings while working in the fields; many participants also reported that text-heavy communications were difficult to understand. Participants appreciated the involvement of personnel from health, agriculture, and environmental sectors, and they recommended expanding such intersectoral collaboration, involving trusted local officials, hiring local actors to help plan and implement the activities, and combining larvicide and fertilizer application in subsequent projects.

**Conclusions:**

Future LSM activities could benefit from social behavior change (SBC) efforts addressing safety and privacy concerns linked to the larvicide and the delivery method and highlight potential health and financial benefits related to decreased malaria burdens in the community, improved quality of life associated with fewer mosquito bites, the efficiency of drones in applying larvicide products, and the need to continue using ITNs. SBC will be most effective if provided early and repeatedly as needed, via channels appropriate to the community’s language and literacy levels and in venues that are geographically and temporally convenient to those potentially impacted by the intervention.

## Introduction

The annual global number of malaria cases has been increasing for several years despite continued investments in vector control [1]. Vector control methods targeting indoor biting, namely insecticide-treated nets (ITNs) and indoor residual spraying (IRS), remain primary methods for malaria prevention. However, limited duration of ITN efficacy [2, 3], emergence of insecticide resistance, mosquitos biting outdoors, and human behaviors limit the impact of these interventions [4, 5]. Supplemental measures to reduce vector populations or control outdoor biting may be required in some settings.

Larval source management (LSM), including use of microbial larvicides, can serve as an adjunct to ITNs or IRS, reducing mosquito populations and dependence on chemical insecticides [6]. Since 2013, WHO has recommended LSM, in addition to ITNs and IRS, where larval habitats are few, fixed and easy to find, such as in desert margins, or in urban, high-altitude, or densely-populated rural areas [7]. Historical concerns about chemical larvicides spurred interest in microbial larvicides, such as *Bacillus thuringiensis israelensis (Bti). Bti* selectively kills mosquito larvae and is safe for non-target organisms, and studies in some sub-Saharan countries have found *Bti*-based larviciding to be generally acceptable and feasible and to reduce malaria vector density [8]. Because of its potential to safely reduce the density of mosquito vectors, *Bti*-based larviciding has been recommended for inclusion in integrated vector management programs, but it is not yet widely implemented for malaria control in Africa [8–10].

Drones, or remotely controlled aircraft, are widely used for recreation, mapping, conservation efforts, emergency response, military purposes, and application of agricultural fertilizers and pesticides because they can travel quickly over difficult or large terrains. In some settings, they have been deployed to spray mosquito larvicides over bodies of water likely to serve as mosquito breeding sites, particularly in rice-growing areas. However, because of their association with military activity and potential safety and privacy concerns, assessing and addressing the acceptability of using drones for public health activities is critical [11–13]. To our knowledge, this has not been done previously in Madagascar.

Madagascar has a high degree of malaria epidemiological, ecological, and climatic heterogeneity; extensive cultivation of rice, which can create “near perfect” mosquito larval habitats [14–16]; evidence of increased outdoor and early-evening biting [17]; and increasing resistance to pyrethroids among malaria vectors [18]. Faced with record increases in malaria in 2021, Madagascar’s national malaria program (NMP) proposed studying the feasibility and entomological impacts of *Bti*-based LSM in two rice-growing districts with relatively high malaria burden and short rainy seasons. As part of that study, we mapped potential vector larval habitats, selected intervention sites near human settlements, and conducted larviciding using drones approximately every two weeks from Feb to July 2022; results of that study will be reported separately. To assess acceptance, perceptions, knowledge, attitudes, and practices around drone-applied, *Bti*-based larviciding in rice fields, we conducted a qualitative study with beneficiary communities in October and November 2022.

## Methodology

### Intervention and setting

Madagascar’s NMP selected Ankazobe District in Analamanga Region and Morombe District in Atsimo-Andrefana Region because of the relatively high malaria burden (annual malaria incidence in 2020 was 106/1,000 in Ankazobe and 101/1,000 in Morombe), the presence of rice fields near human housing, a short rainy season (approximately five months), and receipt of insecticide-treated bednets (ITNs) during the national distribution campaign in 2021. Ankazobe District is centrally located with an average altitude of about 1,100 m; Morombe District is on the southwestern coast with an average altitude of about 40 m. We included six fokontany (small administrative units consisting of up to a few villages) in the district of Morombe, where rice fields are irrigated, and 11 fokontany in the district of Ankazobe, where rice fields are primarily rain-fed, to receive the intervention. The total population in these fokontany was 81,237.

Because the surface area of the rice fields in the intervention areas was vast, we used drones (Agras® T20/T30 unmanned aircraft system) to map and deliver *Bacillus thuringiensis israelensis* (*Bti*) granules (Vectobac® strain AM65-5; Valent BioSciences) dissolved in water to all identified potential vector larval habitats within 1 km of fokontany centers. *Bti* was sprayed at a concentration of about 300 g/hectare approximately every two weeks from February to July 2022, aligning with the rainy rice-growing season, with active spraying lasting about one hour per fokontony per cycle. Each spray cycle was conducted by central-level members of the VectorLink Madagascar project, the drone operating company, and Madagascar’s Ministry of Agriculture and Livestock (MINAE), plus a local social mobilizer (typically the fokontany leader). At the time of this study, drones were rare in Madagascar and were not familiar to the study communities.

Many social and behavior change (SBC) activities were implemented before and during the intervention. Investigators organized courtesy visits to all multi-sectoral governmental and private entities, and with key administrative, religious, and traditional authorities, and non-governmental organizations. Community dialogues were held before spray implementation to inform residents about the intervention and answer their questions. Additionally, the team collaborated with the Ministry of National Education to raise awareness among students and parents; the Ministry of Environment and Sustainable Development (MEDD) to support spray supervision; and the Ministry of Interior and Decentralization to encourage involvement of district chiefs, mayors, and fokontany chiefs for social mobilization. Community mobilizers went door-to-door to distribute flyers, and local radio stations broadcast messages in local dialects about *Bti*, which was described using the term “Bti” and as being a natural, biological product that dissolved easily in water to kill mosquito larvae; the objectives of spraying; the spray schedule; and recommended precautions, such as having people and livestock leave the fields during spraying, one month before the larviciding was initiated and before each spray cycle. SBC activities in Morombe leveraged an association of agricultural water users (Association des Utilisateurs de l'Eau de Bas Mangoky); farmers were able to discuss larviciding activities during association meetings, in addition to the interpersonal communication activities described above.

### Study design

We selected all six intervention fokontany in Morombe and randomly selected seven intervention fokontany in Ankazobe. We recruited participants door-to-door after randomly selecting a house within 1 km of an intervention rice field in each selected fokontany, assessing eligibility (age > 18 years, residence in the fokontany during the LSM campaign), and requesting informed consent. Recruitment proceeded sequentially in the next closest house, regardless of direction, until 18–24 participants had been enrolled in each fokontany. We had intended to recruit and group participants by vocation (rice field owners, rice field workers, and community members who were neither rice field owners or workers) to assess differences in knowledge, attitudes, and perceptions among these groups. However, most adults that we encountered during sampling worked in rice fields, making it difficult to separate these populations into distinct focus groups. We conducted 3 mixed-sex, mixed-vocation focus group discussions (FGDs), each consisting of 6–8 participants, per fokontany, in an attempt to reach data saturation [19].

In-depth interviews (IDIs) were conducted among 17 people from other key groups to help refine FGD guides and add context to the analysis of the FGDs. IDI participants included nurses, doctors, fokontany leaders, municipal leaders, district chiefs, or fokontany notables, and representatives of the MINAE, MEDD, and women’s associations; the project aimed for one representative from each of these groups per district, conveniently selected. The semi-structured interview guide was adapted slightly for each participant type and focused on knowledge of malaria and acceptability and perceptions of (i) the use of *Bti* in rice fields, (ii) drones for the application of larvicides, and (iii) the overall larviciding intervention. IDI data were not formally analyzed because of the small sample size and the risk of breaching confidentiality.

### Data collection and management

The FGDs and IDIs took place between October and November 2022, three to four months after the end of the larviciding, in a quiet setting convenient for the participants.

All FGDs and IDIs were facilitated in Malagasy by a single female moderator and a single female observer/note-taker with previous qualitative research experience. FGDs and IDIs were audio recorded and transcribed verbatim, then translated into French. Study staff assessed the fidelity by listening to the initial 40 min of 4 (10%) recordings and comparing against the transcriptions. Two team members reviewed 4 (10%) pairs of Malagasy and French FGD transcripts for accuracy. Transcripts were not returned to the communities for correction because of cost constraints and limited literacy among participants. FGD transcripts were coded by district, fokontany, and FGD number. Individual responses were given person-level codes during transcription using the observer notes and verbal statement of the individual study identification number by each participant at the start of each FGD.

### Ethical considerations

Participants provided written or, if unable to read or write, verbal consent in the presence of a witness after the consent form was read in Malagasy. To protect participants’ confidentiality, the names of participants, districts, and communities were not included in the interview recordings or reports. The study protocol was deemed non-biomedical research by the Comité d’Ethique et de Recherche Biomédicale à Madagascar (document 109-MSANP/SG/AMM/CERBM) and non-engaged research by the U.S. CDC (project ID 0900f3eb8204991f).

### Data analysis

We analyzed the data using primarily a deductive approach but also allowing emergent themes. French transcripts were first read and coded by the moderator and note-taker. Coded responses were placed in a Microsoft Excel-based analysis grid with five pre-defined topic areas (malaria-related knowledge, and understanding and perceptions of *Bti*, drones, the integrated intervention, and SBC related to the intervention). Codes were compared and discussed until consensus was reached between the two analysts. Subsequently, the transcripts were analyzed to identify additional emergent and cross-cutting themes. We compared and contrasted results across districts and demographic groups, as possible. After initial analysis, additional study team members helped refine themes and develop higher-level themes. IDIs were not formally coded but were read to identify additional probes to add to the FGD discussions, and re-read after FGD transcripts were coded to assess concurrence and contrast with the FGD themes. Participant characteristics were summarized descriptively using Microsoft Excel.

## Results

### Sociodemographic characteristics of respondents

A total of 293 participants took part in the 39 FGDs (21 FGDs and 158 participants in Ankazobe; 18 FGDs and 135 participants in Morombe; Table 1). Of these participants, 120 (41%) were men and 167 (57.0%) had received at least some secondary education. Ethnicities differed by district; most participants in Morombe were Masikoro (75/135; 56%) or Vezo (26/135; 19%) whereas most in Ankazobe were Merina (145/158; 92%). Overall, 69% of participants owned some land used for rice cultivation, with higher proportions of landowners and greater diversity of plot sizes in Ankazobe. Most participants also worked as rice farmers (230/293; 78%). Nearly all (269/293; 92%) participants lived within a 10-min walk of a rice field, and most (253/293; 86%) reported using ITNs to prevent malaria.

**Table 1:** Characteristics of FGD participants

	Morombe		Ankazobe		Total	
	N = 135		N = 158		N = 293	
	n	(%)	n	(%)	n	(%)
*Female*	59	43.7%	61	38.6%	120	41.0%
*Age group, years*						
*18–20*	0	0.0%	2	1.3%	2	0.7%
*21–30*	34	25.2%	51	32.3%	85	29.0%
*31–40*	38	28.1%	34	21.5%	72	24.6%
*41–50*	26	19.3%	28	17.7%	54	18.4%
*51–60*	28	20.7%	25	15.8%	53	18.1%
≥ *61*	9	6.7%	18	11.4%	27	9.2%
*Own a rice field*	64	47.4%	139	88.0%	203	69.3%
*Surface area of owned rice fields*						
*Not applicable*	70	51.9%	18	11.4%	88	30.0%
*0-* < 2 ares*	5	3.7%	14	8.9%	19	6.5%
≥ *2-* < *6 ares*	5	3.7%	40	25.3%	45	15.4%
≥ *6 ares*	55	40.7%	86	54.4%	141	48.1%
*Occupation*						
*Rice farmer*	103	76.3%	127	80.4%	230	78.5%
*Rice farmer only*	41	30.4%	41	25.9%	82	28.0%
*Rice farmer* + *other employment*	62	45.9%	86	54.4%	148	50.5%
*Vegetable or livestock farmer*	5	3.7%	19	12.0%	24	8.2%
*Non-farmer occupation*	27	20.0%	11	7.0%	38	13.0%
*No employment*	0	0.0%	1	0.6%	1	0.3%
*Educational level*						
*No schooling*	14	10.4%	2	1.3%	16	5.5%
*At least some primary schooling*	45	33.3%	65	41.1%	110	37.5%
*At least some secondary schooling*	69	51.1%	87	55.1%	156	53.2%
*At least some university*	7	5.2%	4	2.5%	11	3.8%
*Marital status*						
*Single*	13	9.6%	13	8.2%	26	8.9%
*Married*	103	76.3%	131	82.9%	234	79.9%
*Divorced/Separated*	14	10.4%	8	5.1%	22	7.5%
*Widow*	5	3.7%	6	3.8%	11	3.8%
*Ethnic group*						
*Masikoro*	75	55.6%	0	0.0%	75	25.6%
*Betsileo*	13	9.6%	11	7.0%	24	8.2%
*Merina*	0	0.0%	145	91.8%	145	49.5%
*Mahafaly*	3	2.2%	0	0.0%	3	1.0%
*Vezo*	26	19.3%	0	0.0%	26	8.9%
*Antandroy*	2	1.5%	0	0.0%	2	0.7%
*Other*	16	11.9%	2	1.3%	18	6.1%
*Religion*						
*Protestant*	94	69.6%	50	31.6%	144	49.1%
*Catholic*	41	30.4%	108	68.4%	149	50.9%
*Muslim*	0	0.0%	0	0.0%	0	0.0%
*Walking distance from house to nearest rice field*						
*Near (*< *3 min walk)*	62	45.9%	93	58.9%	155	52.9%
*Far (3–10 min walk)*	66	48.9%	48	30.4%	114	38.9%
*Very far (*> *10 min walk)*	7	5.2%	17	10.8%	24	8.2%
*Malaria prevention methods reported to be used regularly by participant*						
*None*	6	4.4%	20	12.7%	26	8.9%
*ITNs only*	62	45.9%	99	62.7%	161	54.9%
*ITNs and other*†	63	46.7%	29	18.4%	92	31.4%
*Other*	4	3.0%	10	6.3%	14	4.8%

^*^An “are” is 0.01 hectare

^†^”Other” included mosquito coils, burning leaves, and planting vegetation believed to repel mosquitoes

Additionally, 17 participants completed IDIs (nine in Morombe and eight in Ankazobe) (Table 2).

**Table 2:** FGD participants’ knowledge, perceptions, and recommendations related to *Bti*, drones, larviciding activities overall, and larviciding-related social and behavior change activities

Theme	Summary of responses	Illustrative quotes
*Bti*		
*Knowledge*		
Terms for the biolarvicide	In every FGD, some participants volunteered “*Bti”* or “biolarvicide” as the name of the larvicide; however, many participants used other words, primarily “poison,” but also “medicine," "medicine in liquid form," or "earth dust powder," to refer to the product	*The project used* Bti, *but we don't know what it really is… (R19-M-C2-F3-FG1)* *For us here in the rural area, when you talk about a program you're carrying out, we already call it poison. Whether it's medicine or anything else. It's already a habit with us. But in fact, it's a medicine. But here, whatever you bring us is poison. When you spray it on the rice fields, it's called poison*. (R34-M-C1-F1-FG2)
Purpose / mechanism of action	In most FGDs, participants reported that *Bti* is a substance that kills mosquito eggs and larvae. Most participants stated that *Bti* does not kill adult mosquitoes	*It [the drone] sprays the poison that kills insects and mosquito eggs, to prevent the spread of eggs for example in the rice there, or in the ditch there. If the egg dies, the mosquito will not hatch. *(R34-M-C1-F1-FG2) *The larvae consumes the product which kills them but does not kill the adult mosquitoes*. (R11-M-C3-F5-FG1)
***Safety***		
Initial concerns	In most FGDs, participants expressed initial concerns about the effect of *Bti* on crops, fish, and livestock. Seeing the study personnel use personal protective equipment while handling the *Bti* also stoked concerns about its safety	*People agreed [to allow the larviciding], but when the 4* × *4 arrived and the top-of-the-range materials (suit, mask, boots and gloves…), then that's when they said: Ah, ah, ah [meaning no]! It's not appropriate because we're told that the product is biological and harmless.* (R41-M-C2-F4-FG3) *It's better to use organic products because chemical products are… That's why some rice field owners refused. What we knew at first was that it was chemical. And when chemicals are poured into the water, it's really normal if they [farmers] refuse: our fish will die, the rice will be destroyed. Even the environment can be destroyed if chemicals are used.* (R80-A-C4-F7-FG2T) *It's going to cause disease in the cattle if they're feeding in the fields. That was [our thinking] at first. But when they started working, twice, three times, they realized that this thing [Bti] is beneficial.* (R41-M-C3-F5-FG3)
Perceptions at time of FGDs	After additional SBC efforts and observing a lack of harm to people, fish, and livestock, most participants in most FGDs reported feeling that *Bti* was safe	“*It's different from using chemical fertilizers and things like that, and what else? As far as we know, it's harmless. It's like gas, it's odor-free, it's good*.” *(R81-A-C5-F10-FG1)* *At first, people came there [to see the spraying]. Everyone came, assembled and joined them [the study team], and they explain this [the safety and mechanism of action of the Bti].* (R44-A-C6-F11-FG1) *We thought it was the drone that sprayed this disease [bovine diarrhea]. With this thing happening, we made a visit to the doctor [veterinarian] and others. So there is a bovine disease, but it is not the drone that is the cause. It is an epidemic disease unrelated to spraying. It's temporary because everything was affected, even the hamlets where the drone did not spray were affected by the disease, the zebu [cattle] had diarrhea.* (R22 M-C2-F3-FG2) *What's more, it's biological. That's what facilitated the agreement, because it's biological.* (R44-A-C4-F9-FG1)
Drones		
*Safety*		
Initial concerns	Initially, some participants worried about the safety of people and animals near the drones	*It's the drone that's dangerous if you go down into the rice fields during work, not the product [*Bti*].* (R46-M-C1-F4-FG2)
Perceptions at time of FGDs	Over the course of the larcividing activities, participants observed that the drones did not harm people or livestock and most felt that drones were safe to use	*But some say that the work of the drone will destroy our animals, it will kill our farm animals. If we look closely at the result, nothing dies, neither zebu [cattle], nor pig, or duck;, all those who feed in the rice fields have not been killed. So, we would like the continuity of the drone, we have no problem on that.* (R10-A-C4-F7-FG3) *Some rice field owners initially refused to have their fields sprayed by the drone, but accepted after persuasion from those in charge. Then the drone came back and they saw that it was doing a good job, so they finally accepted*. (R34-A-C4-F9-FG2)
*Perceived advantages*		
Efficiency	In all FGDs, participants perceived that using drones to apply larvicide was efficient, allowing rapid coverage of the entire field surface and automation of the spray volume	*It [larviciding] can't be done [by hand]. A person won't be able to spray in the fields. The valley isn't small. That's why he won't be able to do it.* (R59-A-C4-F8-FG1) *The drone drops the right quantity of product without loss.* (R24-M-C3-F5-FG1) *But the work is quick. If he's working in a rice field, for example, let's say he's working for an hour or an hour and a half, he comes back [away from the spraying for a short time] and then he goes back to work. That's what we like about the drone. (R15-M-C3-F6-FG1)* *Rice field workers are paid by the day. So they were not bothered. Officials told them to withdraw from the rice fields from a short time, so they wouldn't have a problem. (R29-M-C2-F4-FG3)*
No-touch application of products	Participants in nearly every FGD appreciated that the drones flew above the crops and did not need to touch them	*It's [the drone is] high up and the rice isn't trampled….For example, if the rice is already producing grains, the person who is going to do it [apply products to the field] will walk between the rice and trample them everywhere. But with the drone, it won't touch the flowers because it'll be flying over them.* (R63-A-C6-F11-FG3) *Ha! It is not good for us, if we directly touch our crops. But this thing (drone) is good for us!” (R80-M-C3-F5-FG2)*
Perceived increase in crop pollination	Many participants believed that wind created by the drones helped with the rice plants’ pollination	*…there's the wind blowing and the rice flower shoots move towards each other and it becomes very productive. The treatment also seems to suit the crop. ( (R27-A-C6-F12-FG1)* *The rice flowers transmit pollen to each other through the effect of the wind [from the drone]. But not that it dies or falls, but it's like a substitute for bees, the wind moves the pollen.* (R47-A-C6-F12-FG1)
Appreciation for use of modern equipment in the community	Many participants reported excitement about seeing the drones being used in their communities; they were interesting to watch and were perceived by a few to increase equity between urban and rural areas	*People in the countryside also want to have modern things like the drone, but not just the people in the city." (R77-A-C6-F12-FG3)* *When the [social] mobilizer arrived at our house, he told us that this was the drone. So everyone looked at it, including the children, because it was something miraculous here in the countryside.* (R61-A-C4-F8-FG3) *People got excited to see the plane [drone] and all that….[It] drew them back….* (R40-A-IDI7)
*Perceived disadvantages and risks*		
Potential crop damage related to drone wind gusts	Some participants, including about half of rice field owners, reported initial concerns about wind gusts from the drones damaging the rice plants, especially during flowering and ripening stages. Many rice field workers suggested flying drones at a greater height when rice is ripe to avoid blowing the grains away. However, many rice field owners and workers reported increased yields despite the lower flight height during the intervention	*It's just that, when the rice starts growing, the drone's flying height seems to be too close. So the plants can't resist the wind, especially when the rice is being born. Maybe there's been a little difference. So if it's feasible, we'd have to take it a little higher than the drone's normal height.* (R35-A-C5-F10-FG1) *Wind, for example from cyclones and other things, destroys the rice during the rice production period. Especially about the drone, there's wind blowing, which can become destructive.* (R32-A-C4-F7-FG1)
Inconveniencing rice field workers	During the IDIs, some participants reported concerns about rice field workers feeling inconvenienced by having to leave the fields during spraying. Rice field workers did not report feeling inconvenienced; a few welcomed the short break during larviciding	*But the workers are paid daily, and that's what they eat with. And when it's cut off for an hour or two, it looks like they're not happy.* (R57-M-IDI3) *Even if, for example, we're going to work on this part of the land, when the technicians arrive, we [rice field workers] move aside a little and come back to it afterwards. There was no impact.* (A-C6-F11-FG1P-R45)
Inconveniencing or frightening passers-by	Some participants, especially those who did not own or work in rice fields, expressed concern about the drones inconveniencing or frightening passers-by	*The drone should not work on market days because there are many people who come from other fokontany [villages] who do not know the drone and might be afraid of it, and also because there are many people who go through the rice fields to go to the market, and this could disturb the work of the drone and pedestrians.* (R37-M-C3-F5-FG3)
Potential for use to spy on or steal resources	Some expressed initial concerns that the drones were being used to locate and assess local resources; however, this concern dissipated after repeated exposure to larviciding activities	*The worries haven't gone away that there's something else involved? Some said the thing [drone] was used to buy the land, he [the drone operator] pretended to look at the zebus [cattle]. So the doubt persisted, whereas later, when he came back often [to apply larvicide], the conscience eased, and we realized that he was bringing good things after all.* (R28-A-C4-F9-FG2)
Potential for use to spread diseases	A few participants reported rumors during the start of the larviciding project about the drones being used to spread diseases such as coronavirus or influenza	*What we thought at the beginning was that it would spray poison that would kill us. Because corona was popular at the time. And so this is going to spray the virus at us. That's what we thought. But it's actually beneficial because it helps us with our health* (R20-M-C2-F4-FG1)
Knowledge and perceived impact of larviciding activities		
Understanding of mosquito lifecycle and habitat	Most participants knew that mosquitoes transmit malaria, and many knew that rice fields can serve as habitat for non-adult mosquitoes	*In my opinion,* Bti *doesn't kill mosquitoes instantly, but it does kill mosquito eggs. Because it's not immediately for today, it's to prevent the future, it's not about killing the living mosquito, it's the egg that's still in the water. The mosquito is still flying around, but the egg it laid is dead. The number of mosquitoes progressively decreases after 1 or 2 sprays, until extermination. When the egg dies, there will be none left, because mosquitoes don't live long.* (R18-A-C4-F7-FG1)
Familiarity with larviciding process	Most participants were very familiar with the larviciding processes	*Here's how the drone works in the rice fields. They stay here, where there are two cars. There's a liquid medicine that they mix in a jar. When it's full, there's a can that they fill, and they place it. They place, they place it on the drone. They start it up. It goes up. It flies like this. It goes, it goes over there, like over there, there's a new commune over there. He goes over there. And he comes back here after a few days, spraying liquid medicine on this area.* (R18-M-C2-F3-FG2)
Perceived value of layering vector control interventions	Some participants reported that larviciding was complementary to ITNs, particularly for those who spent much time in or lived very near the fields	*You can really feel it…even though we were using mosquito nets at the time, because mosquito nets were distributed a long time ago, there was still malaria. But now, there's been an improvement since the drone did the spraying. It has been found that it [malaria] has really decreased. I'll just add that. This mosquito net is still there every year, only those who sleep in the house are covered. But if you go to the field or the yard, you're not protected by the net. We need this effort with the drone.* (R109-A-C4-F9-FG1)
Perceived impact on mosquito density	In nearly all FGDs, participants perceived reductions in mosquito density in the rice fields and their homes	*Then, when the liquid medicine [that] poured from the drone killed those mosquitoes in the field, no more of them came up to the village. That's why there's no more malaria.* (R4-A-C4-F7-FG3) *And now we can sit in the yard, but before we couldn't sit in the yard, when it's evening it's like that. [sound of hands swatting mosquitoes] But now we can sit in the yard. That's the change.* (R42-M-C2-F3-FG3)
Perceived impact on malaria	Most participants perceived fewer malaria illnesses in their community	*In our region, there is also the rice crop season from January to May; that is what is called the rice fever of the new season. This fever when you think about it, is not caused by the rice, but is caused by what happens during the mosquito laying period, which causes the disease. But this time, from January to May, a malaria epidemic period, I didn't hear that people were affected by malaria. Nor that someone or a next door neighbor was taken to the hospital due to malaria. We had not heard much about it.* (R35-A-C6-F12-FG3)
Perceived impact on water purity	In many FGDs in Ankazobe and nearly all FGDs in Morombe, participants perceived improved water quality in the rice fields and an associated reduction in human diarrheal illness	*There are no effects on the human body. Even the water we drink is normal, we don't get stomach aches anymore.*( (R40, R69-A-C4-F8-FG1)
Perceived impacts on rice production	Most participants, especially rice field owners, perceived improvements in rice yield that they attributed to increased pollination due to wind gusts from the drones, and to the Bti acting as a fertilizer and general insecticide	*The rice flowers transmit pollen to each other through the effect of the wind [from the drone]. But not that it dies or falls, but it's like a substitute for bees, the wind moves the pollen.* (R27-47-A-C6-F12-FG1) *And even this [larviciding] improved the color of our rice, it became greener, and the insects that eat the rice all died*. (R15-M-C3-F6-FG1) *It [Bti] acted like a vitamin*. (R23-A-C4-F7-FG3) *But when they started working [larviciding], twice, three times, they realized that this thing [Bti] is beneficial. The rice turned green. (R41-M-C3-F5-FG3)* *And even this improved the color of our rice, it became greener, and the insects that eat the rice all died.* (R15-M-C3-F6-FG1) …[It] was good for both rice and vegetable crops on the same plot of land sprayed by the drone. (R69-A-C4-F8-FG1)
Perceived impacts on finances	Many participants reported increased financial stability related to fewer medical expenses for malaria and diarrhea, fewer missed days of work, and for rice field owners, decreases expenditures on fertilizers and insecticides, all of which they associated with the larviciding	*We would like the drone to continue forever because the money we take to the doctor decreases, and we no longer go to the doctor since the beginning of the spraying.* (R41-M-C1-F2-FG3) *Because I'm healthy, I can work every day.* (R32-M-C3-F5-FG2) *Personally, I'm fine with it because I have a young child. It has had an effect, because my child is protected from malaria. That's why I don't go to the doctor anymore. The fact that I don't spend any money was the best thing for me.* (R16-A-C4-F7-FG3) *If we don't do five to six [fertilizer] treatments, putting products for plants, we cannot hope to get better production. But now, the rice field owners' expenses have decreased because we only do one treatment during the growing period, the production is good.* (R35-A-C4-F8-FG3)
Receipt and perceptions of SBC		
Less useful channels	Posters and displays were less useful because they were text-heavy and most participants could not read well. Radio broadcasts did not reach many participants because most households did not own radios. Community meetings worked well for certain segments but not for rice field workers, who were typically in the rice fields during the meetings	*We [rice field workers] really heard it [messages related to the larviciding], but only in the rice fields, and we can't attend the community meeting*. (R21-M-C3-F3-FG3)
More useful channels	Interpersonal communication and social mobilization via CHWs, local agents of the agricultural department, and engaging village chiefs were viewed as the most successful information-sharing strategy. Most rice field workers reported having been initially informed by rice field owners, and word of mouth was the most commonly recalled channel for subsequent messaging	*…[we saw the] CHW, the village chief, district coordinators with 4* × *4 [a car] and loud speakers. There was a community meeting in the main square, and that's where they provided explanations.* (R42-A-C6-F11-FG3)
Most useful messages	Information they reported viewing as most important included the name and purpose of the larvicide, and the effect of the larviciding on crop yield and the environment	*We already have the name of the drug, which is named after the food of young mosquitoes, which is sprayed.* Bti *is biologic, so there's nothing to worry about because it's made from soil granules. Important to know.* (R11-M-C3-F6-FG2)
**Participants’ explicit recommendations**		
Coordinate across sectors	Some participants suggested additional collaboration between the Ministry of Health and the Ministry of the Environment to find ways to combine larviciding with interventions like fertilizing and applying insecticide	*So I think that if, for example, the Ministry of Agriculture and the Ministry of Health can work together, then* Bti *and fertilizer will come together. Do you see what I mean?* (R72-A-C6-F12-FG3)
Adapt spray timing to local context	A few suggested spraying on days other than market days to avoid inconveniencing or frightening passers-by with the drones	*The drone should not work on market days because there are many people who come from other fokontany who do not know the drone and might be afraid of it, and also because there are many people who go through the rice fields to go to the market, and this could disturb the work of the drone and pedestrians. (R37-M-C3-F5-FG3)*
Increase drone flight height during certain periods of rice plant development	A few suggested raising the drone height to 5 m, especially while plants are very young or after rice grains have formed	*As for the crop, there wasn't much loss of rice. It's just that, when the rice starts growing, the drone's flying height seems to be too close. So the plants can't resist the wind, especially when the rice is being born. Maybe there's been a little difference. So if it's feasible, we'd have to take it a little higher than the drone's normal height. R35-A-C5-F10-FG1)*
Employ community members to participate in larviciding activities	Some suggested hiring community members to participate in the larviciding activities to increase larvicide coverage and generate income within the community	*In my opinion, there will be a double impact [of hiring locals to participate in larviciding activities]. The first impact is that we can fight mosquitoes, the second impact is to be able to give work to people in the surrounding area. (R9-M-IDI6)*
Better adapt SBC channels to local context	Some suggested more visual and non-radio audio messaging help communities understand more easily than text-heavy posters	*If it's about posters, for example. There are fewer posters informing people about the drone. Because here in the countryside, yes, we talk about all the Malagasy there, that when we don't want them to know, we write. But when it's an image, it's easy for people to pick up the information, i.e., they can see right away that this aircraft will be flying over our area. (R41-IDI7)* *But if it's radio, they won't care.(R36-IDI4)*

### *Bti*

#### Knowledge of the product name, purpose, and mechanism of action

In every FGD, at least some participants spontaneously mentioned the terms “biolarvicide” or “*Bti*” when referring to the larvicide used during this project. However, they also used many other terms to refer to *Bti*, such as "*medicine*", "*medicine in liquid form,*" or "*soil dust;*" in every FGD, at least some participants referred to it as "*poison.*" Participants explained that “poison” is used for any product sprayed in agricultural fields, even if it's organic, but typically refers to pesticides. However, the specific term for *Bti* was generally not important to participants, who were more interested in the purpose of *Bti* and its mechanism of action (Table 2).

*For us here in the rural area, when you talk about a program you're carrying out, we already call it poison. Whether it's medicine or anything else. It's already a habit with us. But in fact, it's a medicine. But here, whatever you bring us is poison. When you spray it on the rice fields, it's called poison*. (R34-M-C1-F1-FG2)

In most FGDs (28; 71%) participants reported that *Bti* is a substance that kills mosquito eggs and larvae. Most participants stated that *Bti* does not kill adult mosquitoes.

*In my opinion, Bti doesn't kill mosquitoes instantly, but it does kill mosquito eggs. Because it's not immediately for today, it's to prevent the future, it's not about killing the living mosquito, it's the egg that's still in the water. The mosquito is still flying around, but the egg it laid is dead. The number of mosquitoes progressively decreases after 1 or 2 sprays, until extermination. When the egg dies, there will be none left, because mosquitoes don't live long.* (R18-A-C4-F7-FG1)

#### Evolution of acceptability related to safety concerns

Before larviciding was initiated, the LSM team had disseminated messages about keeping people and livestock out of the fields while spraying was taking place, which contributed to community members’ concerns about the safety of *Bti*. In many FGDs, participants expressed initial concerns about the effect of *Bti* on crops, livestock, and fish, which are an important source of subsistence for these communities. Seeing the study personnel use personal protective equipment while handling the *Bti* also stoked concerns about its safety and limited acceptability. As the larviciding activities began, a bovine diarrhea outbreak occurred in many districts, including in Ankazobe and Morombe, and all focus groups reported initially that a small group of communities perceived that the outbreak was linked to the use of *Bti* in the rice fields.

*People agreed [to allow the larviciding], but when the 4x4 arrived and the top-of-the-range materials [suit, mask, boots and gloves], then that's when they said: Ah, ah, ah [meaning, “no”]! It's not appropriate because we're told that the product is biological and harmless.* (R41-M-C2-F4-FG3)

Safety concerns were reportedly assuaged by social behavior change efforts, including advocacy meetings at the district level and engagement of local veterinarians to deliver messages about the bovine diarrhea outbreak in person and via radio, and participants’ observation of the effects of the larviciding over time. Participants also reported feeling reassured because they could see the *Bti* being mixed into solution on site. At the time of the FGDs, nearly all participants felt that Bti was safe for people, plants, vertebrates, and the environment. That *Bti* is a biologic rather than a chemical product was critical to the participants.

*When people saw that it [*Bti*] didn't destroy anything, then people became grateful.* (R8-M-C2-F4-FG3)

*Before, we thought the product was going to bring us some pain. But* Bti *is not toxic to the rice field workers, who go back to work in the rice fields just after the drone has sprayed.* (R46-M-C1-F4-FG2)

### Drone acceptability

#### Perceived advantages

In all FGDs, participants discussed multiple advantages from using drones to apply larvicide, including efficiency, minimal disturbance of the crops, increased pollination, and a sense of equity and inspiration because very modern technology was used in their communities.

Many participants liked the drones’ ability to rapidly cover the entire field surface (reported to be generally less than an hour per fokontany per application) and to automate the spray volume. Most rice field workers and owners appreciated that the rapid spraying did not meaningfully disrupt their work in the fields or their crop management schedules; this was particularly important when rice field workers were paid by the day. A few participants even suggested that it was pleasant to take a break while the drones were spraying.

*It [larviciding] can't be done [by hand]. A person won't be able to spray in the fields. The valley isn't small. That's why he won't be able to do it.* (R59-A-C4-F8-FG1)

*It can take half a day for someone to work on a rice field. So, half a day lost. But if you use a drone, it's much quicker. What's more, everything will be done. The workers won't mind*. (R38-A-C4-F7-FG3)

Some participants in nearly every FGD appreciated that the drones flew above the crops and did not need to touch them, and workers did not have to walk through the fields.

*It's [the drone is] high up and the rice isn't trampled....For example, if the rice is already producing grains, the person who is going to do it [apply products to the field] will walk between the rice and trample them everywhere. But with the drone, it won't touch the flowers because it'll be flying over them.* (R63-A-C6-F11-FG3)

Over the course of the larviciding activities, many rice field owners and workers came to believe that wind from the drones increased pollination of the rice plants.

*The rice flowers transmit pollen to each other through the effect of the wind [from the drone]. But not that it dies or falls, but it's like a substitute for bees, the wind moves the pollen.* (R47-A-C6-F12-FG1).

Some participants reported satisfaction that drone-based larviciding, which they perceived as a very modern intervention, was being offered to their communities. A few participants perceived that this increased equity.

*When the [social] mobilizer arrived at our house, he told us that this was the drone. So everyone looked at it, including the children, because it was something miraculous here in the countryside.* (R61-A-C4-F8-FG3)

*People got excited to see the plane [drone] and all that….[It] drew them back….* (R40-A-IDI7)

#### Concerns and perceived disadvantages

Many participants reported feeling concerned about drone-based larviciding as it was being introduced. In Ankazobe, participants in a few FGDs spoke about rice field owners having initially declined to have larvicide applied in their fields. Safety was a prominent early concern; some feared that the drones would strike people, livestock, or wild animals. However, over the course of the larviciding activities, most participants perceived no harmful effects in others’ fields and reported beginning to feel that drones were safe to use.

*But some say that the work of the drone will destroy our animals, it will kill our farm animals. If we look closely at the result, nothing dies, neither zebu [cattle], nor pig, or duck; all those who feed in the rice fields have not been killed. So, we would like the continuity of the drone, we have no problem on that.* (R10-A-C4-F7-FG3)

Additional early concerns among some participants, including about half of rice field owners, centered on the potential for wind gusts from the drones damaging the rice plants, especially during flowering and ripening stages. Some rice field owners suggested flying the drones at a greater height while rice plants are immature, and many rice field workers suggested flying drones at a greater height (such as 5 m) when rice grains were nearly ripe to avoid blowing the grains away.

*It's just that, when the rice starts growing, the drone's flying height seems to be too close. So the plants can't resist the wind, especially when the rice is being born. Maybe there's been a little difference. So if it's feasible, we'd have to take it a little higher than the drone's normal height.* (R35-A-C5-F10-FG1)

During the IDIs, some participants reported concerns about rice field workers feeling inconvenienced by having to leave the fields during spraying. One participant suggested spraying later in the afternoon, when workers would have completed their work, or to inform the workers of the spray schedule in advance. However, nearly all related comments from rice field workers suggested that they did not mind needing to leave the fields briefly during spraying.

*… [I] suggested spraying in the afternoon, when there are fewer people, and to let the workers know the schedule, so that they can work and retire for the 30 minutes when the drone sprays the plot and come straight back to work right afterwards.* (R4-M-IDI5)

*But the workers are paid daily, and that's what they eat with. And when it's cut off for an hour or two, it looks like they're not happy.* (R57-M-IDI3)

Some participants, particularly participants who did not own or work in rice fields, raised concerns about inconveniencing passers-by with dust and wind generated during the drones’ take-off and landing. A few worried that others traveling to the area on market days may be afraid of the drones.

*The drone should not work on market days because there are many people who come from other fokontany [villages] who do not know the drone and might be afraid of it, and also because there are many people who go through the rice fields to go to the market, and this could disturb the work of the drone and pedestrians. (*R37-M-C3-F5-FG3)

Some expressed initial concerns that the drones were being used for nefarious purposes, such as to locate and assess local resources such as minerals, gold, and cattle or to spread diseases like COVID-19 and influenza. However, over the course of the five months of larviciding, participants developed trust in the larviciding teams and activities.

*The worries haven't gone away that there's something else involved? Some said the thing [drone] was used to buy the land, he [the drone operator] pretended to look at the zebus [cattle]. So the doubt persisted, whereas later, when he came back often [to apply larvicide], the conscience eased, and we realized that he was bringing good things after all.* (R28-A-C4-F9-FG2)

### Overall knowledge and perceptions of larviciding

#### Familiarity with larviciding processes

In nearly all FGDs, participants were very familiar with the larviciding process.

*Here's how the drone works in the rice fields. They [drone operators] stay here, where there are two cars. There's a liquid medicine that they mix in a jar. When it's full, there's a can that they fill, and they place it. They place, they place it on the drone. They start it up. It goes up. It flies like this. It goes, it goes over there, like over there, there's a new commune over there. He goes over there. And he comes back here after a few days, spraying liquid medicine on this area.* (R18-M-C2-F3-FG2)

*It's a small plane that sprays every 15 days and kills mosquito eggs.* (R44-M-C3-F5-FG2)

#### Perceived impacts on mosquitoes and malaria

In 35 (90%) FGDs, many participants perceived that the larviciding decreased mosquito density in rice fields, homes, and yards. This was reported to increase their ability to relax outdoors without being bitten by mosquitoes. Some participants expected larger decreases in the numbers of non-vector, nuisance mosquitoes (“short mosquitoes”) but were satisfied by perceived reductions in the numbers of malaria-carrying mosquitoes. Most participants also perceived fewer malaria cases. Some perceived greater impacts from larviciding than from other vector control interventions, such as ITNs, and advocated layering these interventions.

*And now we can sit in the yard, but before we couldn't sit in the yard, when it's evening it's like that. [sound of hands swatting mosquitoes] But now we can sit in the yard. That's the change.* (R42-M-C2-F3-FG3)

*You can really feel it, because since then, malaria has been very widespread, but even though we were using mosquito nets at the time, because mosquito nets were distributed a long time ago, there was still malaria. But now, there's been an improvement since the drone did the spraying. It has been found that it has really decreased. I'll just add that. This mosquito net is still there every year, only those who sleep in the house are covered. But if you go to the field or the yard, you're not protected by the net. We need this effort with the drone.* (R109-A-C4-F9-FG1)

#### Perceived impacts on production of rice and other crops

Most participants, particularly rice field owners, perceived improved rice yields following the larviciding. They perceived taller and greener rice plants, which they believed resulted from the *Bti* killing insects that are harmful to rice plants, such as butterfly larvae and rice lice, and from *Bti* acting as a “vitamin” or fertilizer for the plants. A few reported positive impacts on their non-rice crops growing near the rice fields.

*And even this improved the color of our rice, it became greener, and the insects that eat the rice all died*. (R15-M-C3-F6-FG1)

*It [Bti] acted like a vitamin*. (R23-A-C4-F7-FG3)

However, most rice field workers reported that the weather was the primary factor impacting yield.

*But in terms of non-productivity, it depends on the weather, but it doesn't depend on the drone. It does not depend on the drug [Bti].* (R33-A-C6-F11-FG1)

#### Additional perceived impacts on health, finances, and quality of life

In about two-thirds of FGDs, and in nearly all the FGDs among rice field workers in Morombe, participants reported perceiving improved water quality in the rice fields and less diarrhea among community members.

*Even the water we drink is normal, we don't get stomachaches anymore.* (R40, R69-A-C4-F8-FG1)

Many participants reported fewer days of work missed because of illness; increased financial stability because of increased crop yields and fewer expenses for medical care (due to less malaria and diarrhea), fertilizers, and insecticides; and more pleasant outdoor experiences at home and while working in the rice fields because of fewer mosquito bites.

*Because I'm healthy, I can work every day.* (R32-M-C3-F5-FG2)

*If we don't do five to six [fertilizer] treatments, putting products for plants, we cannot hope to get better production. But now, the rice field owners' expenses have decreased because we only do one treatment during the growing period, the production is good.* (R35-A-C4-F8-FG3)

#### Receipt and perceptions of larviciding-related SBC

Some participants from the general community mentioned having seen displays or posters about the larviciding activities, but since they could not read, images of the drones primarily attracted their attention. Most participants did not hear the project’s radio broadcasts because few participants’ households owned radios. A few participants said they heard about the larviciding from other community members, but they did not attend the community meetings themselves. Interpersonal communication and social mobilization via CHWs, local agents of the agricultural department, and village chiefs were viewed as the most successful information-sharing strategy because they were already accustomed to raising community awareness and were familiar to community members. Because rice field workers generally spend all day in the fields and cannot attend community meetings, nearly all rice field workers reported having been informed of the larviciding by the rice field owners and receiving and spreading the news through word of mouth.

*…[we saw the] CHW, the village chief, district coordinators with 4x4 [a car] and loud speakers. There was a community meeting in the main square, and that's where they provided explanations.* (R42-A-C6-F11-FG3)

*We [rice field workers] really heard it [messages related to the larviciding activities], but only in the rice fields, and we can't attend the community meeting*. (R21-M-C3-F3-FG3)

In nearly all FGDs, participants reported that they received sufficient information before the larviciding activities began. Information they reported feeling was most important included the name and purpose of the larvicide, and the effect of the larviciding on crop yield and the environment.

*We already have the name of the drug, which is named after the food of young mosquitoes, which is sprayed. Bti is biologic, so there's nothing to worry about because it's made from soil granules. Important to know.* (R11-M-C3-F6-FG2)

#### Participants’ explicit recommendations for future larviciding activities

Some participants suggested further collaboration between ministries of health, agriculture, and environment to combine *Bti* larvicide with fertilizers to further increase crop yield. Some also suggested hiring community members to spray larvicide via backpack sprayers in hard-to-reach areas and ponds, canals, wells, and other areas of stagnant water, as an adjunct to drone-delivered larviciding in the rice fields.

*In my opinion, there will be a double impact [to hiring local people to work on future larviciding activities]. The first impact is that we can fight mosquitoes, the second impact is to be able to give work to people in the surrounding area. (R9-M-IDI6)*

Additional recommendations from some participants included spraying on non-market days to avoid inconveniencing or frightening passers-by with the drones; raising the drone flight height to 5 m, especially while plants are very young or after rice grains have formed; and adding more visual and audio messaging to the SBC package.

*Conduct awareness campaigns in the local dialect, so that the whole community can understand the messages. Make awareness-raising videos to circulate in the fokontany to help communities understand more easily with images: the eyes watch, ears listen, and reflection continues. (M-C1-F1-FG3C R33)*

## Discussion

Rice fields are an important source of malaria vectors [15, 16], and understanding the perceptions and acceptability of larviciding in rice agricultural settings, perhaps particularly when drones are involved, has been identified as a critical information gap [11–13, 15]. In this study of knowledge and perceptions of larviciding that had been delivered free of charge to rice fields in two districts in Madagascar, participants across sites and demographic groups were highly accepting of *Bti*-based larviciding and the use of drones to apply it. The detailed insights, perceptions, and concerns from the present study could help optimize future malaria vector control interventions and associated SBC activities and provide insights into how drones may be perceived when used for other health interventions.

### *Bti*

Although several studies have shown the safety of biolarvicides in the environment [8, 20], initial concerns about *Bti* safety threatened acceptance of the intervention. Some participants initially believed the gloves, gowns, and masks worn by study staff during handling of the *Bti* meant that the product was toxic. Ultimately, following additional SBC efforts and participants’ observations that ill effects did not occur, these communities generally perceived *Bti* to be safe for humans, crops, livestock, and the environment. This perception was an important factor in the acceptance of *Bti*-based larviciding. Participants in Madagascar also strongly appreciated that *Bti* was a biological, rather than a chemical, product; they felt that biological products are safer than chemical products, despite also using terms like “medicine” and “poison” in place of “*Bti*.” Similar positive perceptions of *Bti* safety for larviciding have been reported in Tanzania, where “all participants reported to trust that bio larvicide used had no effects on human, field and animal health,” and in Rwanda [21, 22]. However, in another study in Rwanda, around 30% of community members believed *Bti* to be unsafe for agricultural products and farmers or miners [23].

Participants demonstrated good, basic understanding of malaria transmission, mosquito habitats and life cycles, and the anticipated role of the *Bti*, which appeared to help with understanding and acceptance of larviciding. The communities in the study sites had received related SBC before and during the intervention, which may have improved their level of knowledge about these factors. Setting expectations about the magnitude of impact on mosquito populations may help further improve the acceptability of future larviciding activities, as persistence of nuisance mosquitoes near participants’ homes caused confusion or disappointment among at least some participants.

### Drones

Limited data exists about the acceptability of drones for public health activities in Sub-Saharan Africa. Formative research in Zanzibar found that community members would support the use of drones for public health purposes provided they were informed of the plans and purposes before implementation began, and if community leaders consented [12]. In Rwanda, more than 80% of farmers and miners perceived drones as safe or very safe for larviciding, while 11% perceived them to be unsafe [23]. Frameworks for planning to use drones for vector control activities advise addressing public safety and privacy concerns by following local civil aircraft regulations, regularly maintaining drones and training operators, organizing flight timing and paths to be minimally disruptive to residents, and meeting with local stakeholders during planning and implementation phases to understand and mitigate their concerns [12, 13]. In Madagascar, participants shared many safety and privacy concerns about using drones to apply larvicide. Initially, a few participants worried that the drones could be used to disperse human pathogens or poisons intended to harm the community; some also expressed concerns that the drones were being used to locate and potentially steal local resources, like land and cattle; and many participants initially feared that the drones would strike people in the vicinity, intentionally or unintentionally. Participants reported that these fears were primarily allayed by observing that no ill effects occurred after repeated cycles of larviciding in their communities. However, formative investigation of local concerns and earlier community engagement would have helped better tailor SBC before and during larvicidng activities to allay these concerns and potential rumors more quickly [12, 15].

Participants also perceived several benefits to using drones to apply larvicide. Manual rice field larviciding in Rwanda was associated with concerns about time commitments, slippery terrain, and inability to treat the centers of large fields [21]; participants in Madagascar endorsed efficiency and less trauma to rice plants while using drones, compared to manual spraying, and some believed that the wind from the drones improved rice pollination. Concerns remaining at the time of the FGDs included drone-derived wind gusts potentially damaging young rice plants or blowing away mature rice grains, although many participants also perceived higher rice yields during the season larviciding took place. Many participants worried about the drones inconveniencing or frightening passers-by; they suggested timing the drone flights to avoid market days.

### Perceptions of intervention delivery and impact

As in other studies, participants widely perceived that the larviciding decreased mosquito density and biting in rice fields, homes, and yards [21, 22, 24]. Results of entomological monitoring in the intervention and control sites in Madagascar are not yet available to corroborate or refute this perception; however, studies elsewhere have measured decreased abundance of adult mosquitoes following *Bti*-based larviciding [8].

Although data on rice yields in the intervention and comparison areas were not collected as part of this study, many participants perceived an increase in rice yields, in contrast to the perceptions following *Bti*-based larviciding in Rwanda [21]. Collaborating with agronomists to better understand whether and how larviciding affects yields may be useful in future studies [15].

The larviciding activities in Madagascar were centrally managed and provided free of charge to the communities. In other settings, rice field owners suggested that strong community ownership of larviciding activities, including implementation via local rice cooperatives, would help increase acceptability and decrease concerns about safety and privacy [21, 25]. Participants in other studies also endorsed community financing alongside government or partner financing [21, 24], and in a study of Rwandan farmers and miners, > 80% were willing to contribute financially and > 90% were willing to provide at least 1 h of labor biweekly for larviciding [23]. Building local capacity to implement and manage the activities may require substantial investment but would help ensure acceptability, sustainability, and equity [8, 26].

While not explicitly mentioned by the study participants in Madagascar, larviciding activities may lead to decreased perceived need to use ITNs [21]. Regular ITN use was reported by only 81% of participants in Ankazobe district, suggesting that programs should consider integrating ITN- and larvicide-related social and behavioral change activities.

### SBC messaging content and channels

Early awareness-raising efforts included multiple trusted stakeholders from the national and local levels, and multiple modes of engagement (posters, community meetings, person-to-person), both of which are considered critical to the acceptability of LSM [10]. However, some important concerns, such as fears that the drones would disperse human pathogens, had not been identified or addressed before larviciding was started; these could have threatened the project. Engagement with SBC activities appeared to differ by demographic group, with rice field workers frequently reporting having missed community meetings while working in the fields; many participants also reported that text-heavy communications were difficult to understand. Earlier and more tailored social and behavior change activities could further reduce the risk of rumors and increase acceptability of future larval source management activities [10].

## Limitations

The findings we report may not be representative of other rice-growing districts in Madagascar, or representative of the general population in the rice-growing villages in these districts. Although we had planned separate FGDs for rice field owners and workers and the general population to understand various perspectives, we found that most households in the target communities owned some agricultural land or worked in rice fields, making it difficult to organize separate vocation-based FGDs. This aligned with the results of the 2020 Madagascar census, which found that nearly 80% of households engaged in agricultural activities [27]. Next, because we purposefully included rice field owners, who are mostly men, women’s perspectives were slightly less prevalent in the FGDs. The FGDs and IDIs were conducted at least three months after the intervention had concluded, and participants might have forgotten some of their concerns and perceptions. Data were translated from Malagasy to French to English, which might have caused some changes in nuance, although quality checks were performed on the translations. Finally, a national insecticide-treated bed net campaign was conducted in late 2021. Malaria burden decreased substantially across Madagascar in 2022, without a significant difference in intervention and comparison areas in these two districts [28]. This might have biased perceptions of the effectiveness of the larviciding.

## Recommendations and conclusions

We found that both *Bti*-based larviciding and using drones to apply it were ultimately well accepted by rice field owners and workers and community members in rice-growing areas of Madagascar, despite some initial unease. Key concerns spanned safety of *Bti* for people, livestock, and the environment, and harms related to drones crashing or being used for nefarious purposes. Participants suggested close intersectoral collaboration, involvement of trusted local officials, hiring local actors for win–win economic benefits, and combining larvicide and fertilizer application in subsequent projects. Future plans for *Bti-*based or other larviciding should include clear communication between trusted partners and beneficiary communities about the nature of the product, its mechanism of action, its safety profile, and the rationale for staff using personal protective equipment, before implementation begins and as needed during implementation [25]. SBC activities could also address privacy concerns and highlight potential health and financial benefits related to decreased malaria burdens in the community, improved quality of life associated with fewer mosquito bites, the efficiency of drones in applying larvicide products, and the need to continue using ITNs. Related communications will be most effective if tailored to the community’s language and literacy level, provided through trusted channels in venues that are geographically and temporally convenient to those potentially impacted by the intervention, and adapted and continually updated to address specific local concerns [12, 13, 15].

While this study focused on the acceptability of drone-applied *Bti* larvicide in rice fields, larviciding is only one potential method of LSM that could be used in rice fields. Other LSM interventions could include environmental management or environmental modification of rice fields [10, 29], and some lessons from the present study could be relevant. For example, the multisectoral and multi-ministerial approach in Madagascar conferred a number of advantages, such as participants receiving communications and messaging from multiple, trusted partners in the agricultural and education sectors; incorporation of environmental considerations from agricultural water use associations and the Ministry of the Environment; and mobilization of district chiefs, mayors, and fokontany chiefs by the Ministry of Interior and Decentralization to increase trust, awareness, and coordination. These approaches were critical in garnering acceptance of drone-delivered, *Bti*-based larviciding in this study and could be considered to help bolster acceptance and local leadership for other methods of LSM in rice agriculture settings.

## Data Availability

The study focus group discussion transcripts are available on request.
